# Alteration of skin perfusion in mottling area during septic shock

**DOI:** 10.1186/2110-5820-3-31

**Published:** 2013-09-16

**Authors:** Hafid Ait-Oufella, Simon Bourcier, Mikael Alves, Arnaud Galbois, Jean-Luc Baudel, Dimitri Margetis, Naike Bige, Georges Offenstadt, Eric Maury, Bertrand Guidet

**Affiliations:** 1 Service de réanimation médicale, Hôpital Saint-Antoine, Assistance Publique-Hôpitaux de Paris, 184 rue du Faubourg Saint-Antoine, Cedex 12, Paris 75571, France; 2 Paris Research Cardiovascular Center, Inserm U970, Paris, France; 3 Université Pierre et Marie Curie-Paris 6, Paris, France; 4 Inserm U707, Paris F-75012, France

**Keywords:** Septic shock, Microcirculation, Mottling, Intensive care medicine

## Abstract

**Background:**

Mottling score has been reported to be a strong predictive factor during septic shock. However, the pathophysiology of mottling remains unclear.

**Methods:**

In patients admitted in ICU for septic shock, we measured on the same area the mean skin perfusion by laser Doppler, the mottling score, and variations of both indices between T1 (6 hours after vasopressors were started) and T2 (24 hours later).

**Results:**

Fourteen patients were included, SAPS II was 56 [37–71] and SOFA score at T1 was 10 [7–12]. The mean skin surface area analyzed was 4108 ± 740 mm^2^; 1184 ± 141 measurements were performed over each defined skin surface area. Skin perfusion was significantly different according to mottling score and decreased from 37 [31–42] perfusion units (PUs) for a mottling score of [0–1] to 22 [20–32] PUs for a mottling score of [2–3] and 23 [16–28] for a score of [4–5] (Kruskal-Wallis test, *P* = 0.05). We analyzed skin perfusion changes during resuscitation in each patient and together with mottling score variations between T1 and T2 using a Wilcoxon signed-rank test. Among the 14 patients included, mottling score increased (worsened) in 5 patients, decreased (improved) in 5 patients, and remained stable in 4 patients. Baseline skin perfusion at T1 was arbitrarily scored 100%. Mean skin perfusion significantly decreased in all the patients whose mottling score worsened from 100% baseline to 63.2 ± 10.7% (*P* = 0.001), mean skin perfusion significantly increased in all patients whose mottling score improved from 100% baseline to 172.6 ± 46.8% (*P* = 0.001), and remained stable in patients whose mottling score did not change (100.5 ± 6.8%, *P* = 0.95).

**Conclusions:**

We have shown that mottling score variations and skin perfusion changes during septic shock resuscitation were correlated, providing additional evidence that mottling reflects skin hypoperfusion.

## Background

The identification of endothelial dysfunction and abnormal microcirculation as the main cause of organ damage and death during septic shock [1,2] prompts intensivists to develop tools to assess microcirculation and organ perfusion. Sublingual videomicroscopy using Sidestream Dark Field (SDF) imaging provided interesting informations on microcirculatory status during septic shock at the admission and during resuscitation with a direct visualisation of microvessels [3]. Noninvasive measurements of tissue oxygenation using near-infrared spectroscopy (NIRS) technology informs indirectly about microcirculation and mostly muscle perfusion [4]. In our unit, we focused on skin perfusion through the exploration of mottling. We have developed a clinical score based on the extension of mottling around the knee and showed in a prospective, observational study that mottling score was a strong predictor of 14-day mortality for patients admitted for septic shock. Mottling score was related to other parameters that reflected organ perfusion, such as arterial lactate level or diuresis [5]. However, the physiopathology of mottling remains unknown, and the link between mottling and skin perfusion was speculative and based on indirect evidences. To explore in a more accurate way the physiopathology of mottling, we conducted a pilot observational study using a laser Doppler imager to analyze skin perfusion according to mottling extension during septic shock management.

## Methods

### Septic shock inclusion and management

We conducted a prospective, observational study in a 16-bed ICU in a tertiary teaching hospital during 4 months. All consecutive patients, older than 18 years, admitted for septic shock were included. Septic shock, within 24 hours after ICU admission, was defined according to the 2001 SCCM/ESICM/ACCP/ATS/SIS International Sepsis Definitions conference [6]. Patients were included (H0) when vasopressor infusion was started within 24 hours of admission. Circulatory support was guided by our local protocol, adapted from international guidelines [7]. Intravenous volume expansion and intravenous norepinephrine were used in a stepwise manner to achieve predefined endpoints of resuscitation from invasive hemodynamic monitoring: mean arterial pressure (MAP) >65 mmHg, central venous pressure (CVP) between 8 and 12 mmHg, urinary output >0.5 ml/kg/h. General characteristics of the patients were recorded: demographic data, diagnoses, severity of illness evaluated by the Sequential Organ Failure Assessment (SOFA) score (within 6 hours of admission) [8] and Simplified Acute Physiology Score II (SAPS II) [9]. Hemodynamic variables were recorded 6 hours (T1) and 24 hours (T2) after start of vasopressor therapy. We measured MAP, CVP, diuresis, cardiac index using echocardiography, arterial lactate level, and mottling score. Mottling score, recently described by our group, provided a semiquantitative evaluation of mottling based on skin area extension on legs. Score 0 no mottling, score 1 small mottling area (coin size) localized to the center of the knee, score 2 mottling area that does not exceed the superior edge of the knee cap, score 3 mottling area that does not exceed the middle thigh, score 4 mottling area that does not exceed the fold of the groin and score 5 otherwise [5]. Finally, we measured mean skin perfusion using Laser Doppler Imager on the legs and compare its changes to mottling score variations between T1 and T2.

### Scanning laser Doppler

The PeriScan PIM 3 System is based on the established laser Doppler technique [10,11]. When laser light penetrates the tissue, it is scattered and partly absorbed. Some of the scattered light returns to the tissue surface, where it is registered by a photo detector inside the instrument. This signal is then processed to extract information about the microcirculatory blood flow. According to the Doppler principle, light particles which hit moving blood cells undergo a change in wavelength/frequency (a Doppler shift), while light particles which encounter static structures return unchanged. The perfusion can be calculated, because the magnitude and frequency distribution of the Doppler shifted light are directly related to the number and velocity of blood cells. Blood perfusion is measured in perfusion units (PU) and there is a documented linearity between PU and the true blood perfusion in the tissue being imaged. The penetration depth is between 0.5 and 1 mm. The PeriScan PIM 3 System automatically scans a defined skin surface area (4 milliseconds per pixel, resolution of 255 × 255 pixels). The data are computed and visualized as a two-dimensional colour-coded image, mapping skin perfusion. The mean blood flow is computed to yield an average of pixels in a region of interest within the scanned area with the software provided by the manufacturer (LDPIwin software). The total time required for a complete image at these settings is approximately 5 minutes. The scanning Laser Doppler provides a skin perfusion mapping and the LDPIWin software led us to measure skin perfusion, expressed in Perfusion Units (PU), in the same area that we quantified the mottling score.

### Study protocol

**Figure 1:**
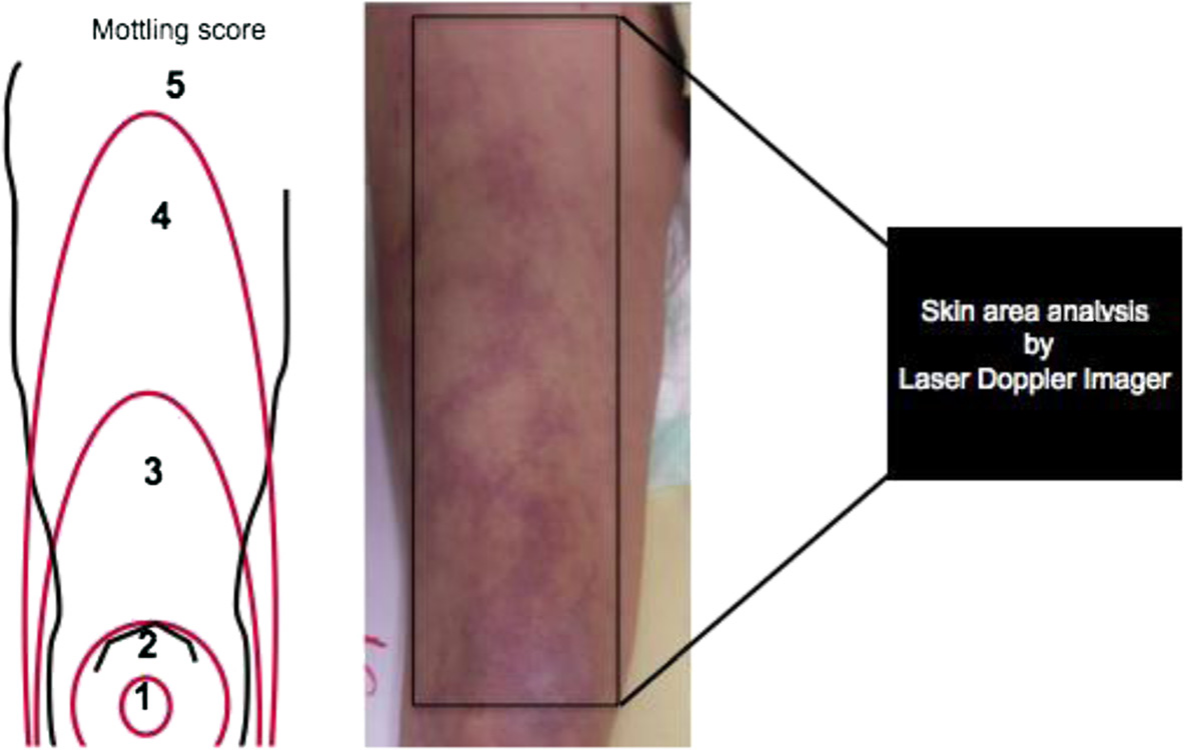
**Mottling score is based on mottling area extension on legs.** Score 0, no mottling; score 1, modest mottling area (coin size) localized to the center of the knee; score 2, moderate mottling area that does not exceed the superior edge of the kneecap; score 3, mild mottling area that does not exceed the middle thigh; score 4, severe mottling area that does not exceed the fold of the groin; and score 5, extremely severe mottling area that exceeds the fold of the groin. The area of laser Doppler scanning was superposed to the area of mottling score classification.

Experiments were performed at bedside. The first exploration (T1) was done 6 hours after vasopressor infusion has been started and the second 24 hours later (T2). The laser Doppler imaging was performed with the patient in a reclined position, the thigh at heart level. The distance between the skin and the lowest part of the scanner head during image capturing was fixed to 17 cm with the system resolution set on medium. Studied skin area was standardized and was focused on the anterior face of the thigh between the middle of the knee cap and the fold of the groin (Figure 1). When capturing images, the ambient light level was kept at the minimum to avoid any influence on the laser light and the quality of the recorded perfusion signal. We measured mean skin perfusion at T1 and T2, and we also compared skin perfusion changes with mottling score variations between T1 and T2. The investigator that analyzed the data provided by the laser Doppler imager was blinded to the mottling score.

The observational protocol was approved by the ethical committee of the *Société de Réanimation de Langue Française (SRLF).* This is an observational study without any specific intervention. All patients and families were informed through the admission leaflet that anonymous data could be used for academic research and gave their consent.

### Statistical analysis

Data were summarized as median (25^th^-75^th^ percentiles) for skewed distributions and percentages as appropriate. Skin perfusion expressed on PUs according to mottling score was tested using the Kruskal-Wallis rank-sum test. Hemodynamic variables and skin perfusion changes between T1 and T2 were tested using a Wilcoxon signed-rank test. All tests were computed with the R software. Significance was defined as a two-sided *P* value < 0.05.

## Results

### Studied population

During the 4-month study, fourteen patients were included. General characteristic of the studied population were summarized in Table 1. The median SAPS II was 56 [37–71], the median SOFA at T1 was 10 [7-12], and the 14-day mortality was 36% (5/14 patients). All the patients initially received intravenous norepinephrine; median dose was 0.20 μg/kg/min [0.10-0.37] at T1 and 0.11 μg/kg/min [0.01-0.72] at T2. Hemodynamic parameters at both T1 and T2 were summarized in Table 2.

**Table 1:** Baseline characteristics of included population

**Patients (n)**	**14**
Age, yr	65 [53–68]
Gender, female (n)	5/14
Primary site of infection [n]	
Lung	3
Abdomen	6
Urinary tractus	1
Endocarditis	1
Primary bactaeremia	3
SAPS II	56 [37–71]
14-day mortality (n)	5/14

SAPS II (Simplified Acute Physiology Score) was calculated within 24 hours of admission.

Values are given as median [25^th^-75^th^ percentiles] and numbers (n).

**Table 2:** Hemodynamic parameters recorded at T1 (6 hours after vasopressor start) and T2 (24 hours after vasopressor start)

	**T1**	**T2**	***P***
SOFA score	10 [7–12]	11 [3–16]	NS
Norepinephrine			
N	14	10	
Doses μg/kg/min	0.20 [0.10-0.37]	0.25 [0.01-0.72]	NS
Mean arterial pressure (mmHg)	77 [66–88]	84 [72–91]	NS
Central venous pressure (mmHg)	13 [10–15]	12 [11–16]	NS
Cardiac index (L/min/m2)	2.4 [2.2-2.9]	2.3 [2.1-3]	NS
Arterial lactate level (mmol/L)	3.3 [1.8-7.2]	1.8 [1.2-6]	NS
ScVO_2_ (%)	70 [67–75]	71 [67–77]	NS

*SOFA* Sequential Organ Failure Assessment, *ScVO2*, central venous saturation in oxygen, *NS* not significant.

Values are given as median [25^th^-75^th^ percentiles]. Cardiac index was measured using echocardiography.

### Laser Doppler analysis

**Figure 2:**
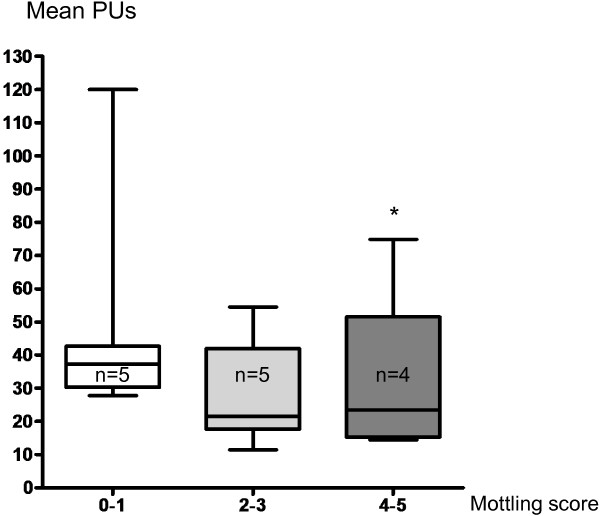
**Mean skin perfusion expressed as mean perfusion units (PUs) according to mottling score.** Boxes show 1st and 3rd quartiles, with the median as a thick line. Whiskers extend to 1.5 interquartile ranges (Q75-Q25). *P = 0.05.

**Figure 3:**
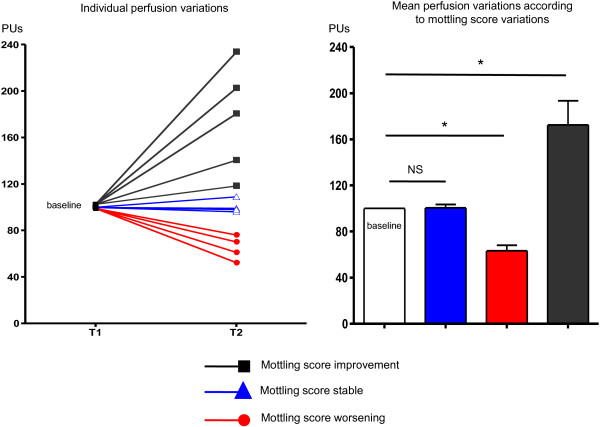
**Left: individual skin perfusion changes between T1 and T2.** Baseline value was 100%. Right: mean skin perfusion changes between T1 and T2 according to mottling score variations. Baseline value was 100%. *P < 0.05, **P < 0.01.

The mean skin surface area analyzed was 4108 ± 740 mm^2^; 1184 ± 141 measurements were performed over each defined skin surface area. First of all, we compared the mean skin perfusion according to mottling score at T1. Interestingly, mean skin perfusion was significantly different according to mottling score and decreased from 37 [31–42] PUs for a mottling score of [0–1] (n = 5) to 22 [20–32] PUs (n = 5) for a mottling score of [2,3] and 23 [16–28] for a score of [4,5] (n = 4) (*P* = 0.05) (Figure 2). However, value variability was important because PU depends on several individual parameters such as microvessel density or skin thickness. For this reason, we subsequently analyzed the skin perfusion changes during resuscitation in each patient and compared the results to mottling score variations between T1 and T2. Among the 14 patients included, mottling score increased (worsened) in 5 patients, decreased (improved) in 5 patients and remained stable in 4 patients. Baseline value at T1 was noted 100%. Interestingly, we observed that mean skin perfusion significantly decreased in all the patients whose mottling score worsened from 100% baseline to 63.2 ± 10.7% (*P* = 0.001), mean skin perfusion significantly increased in all the patients whose mottling score improved from 100% baseline to 172.6 ± 46.8% (*P* = 0.02), and remained stable in patients whose mottling score did not change (100.5 ± 6.8%, *P* = 0.91; Figure 3).

## Discussion

The identification and the quantification of microcirculation dysfunction during severe infection is a major challenge in ICU [1,12]. We focused on a very old clinical sign of shock, mottling [13,14], and hypothesized that mottling was the consequence of abnormal skin perfusion. To quantify mottling, we developed a clinical score based on its skin extension over the leg. This score, easy to learn and to use at the bedside, is very reproducible (kappa 0.87, 95% confidence interval (0.72-0.97)) [5]. In a prospective, observational study, we have shown that mottling score and its variations during septic shock resuscitation provides powerful predictive information. Moreover, mottling score was strongly related to parameters (lactate level or urinary output) that reflect tissue perfusion [5].

However, the physiopathology of mottling remains unknown. Several indirect evidences led us to associate mottling and abnormal skin perfusion. First, mottling areas are colder than normally colored skin (personal observations). Moreover, we have recently reported, using NIRS Technology, that tissue oxygen saturation on knee area was inversely related to mottling score, the higher the mottling score the lower the knee StO_2_[15]. In healthy volunteers, Lima et al. also reported that peripheral vasoconstriction induced by body surface cooling reduced StO_2_[16]. As StO_2_ measures tissue oxygen saturation within 15 mm of depth that included skin and muscle, it could be an argument for abnormal skin perfusion in mottling area but the respective part of both tissues (skin and muscle) in the final result of stO_2_ is unknown. In this pilot study, we evaluated the link between mottling extension and skin perfusion. The skin perfusion was measured using a scanning laser Doppler (PIM3 Perimed System) on the same area that we quantified the mottling score (Figure 1). We measured mean skin perfusion with more than 1,000 measurements, and we observed that mean skin perfusion was inversely related to mottling score. Distribution of the mean perfusion values between patients was large, because skin perfusion depends on several individual parameters, such as microvessels density, skin thickness, hemoglobin level, and skin temperature. Between T1 and T2, we did not observe any difference regarding the central temperature or the hemoglobin level. Between T1 and T2, we observed three different profiles, a diminution (improvement) of mottling score in five patients, an augmentation (worsening) in five patients, and no variation in four patients. Mottling score changes during resuscitation are very informative, because we have previously shown in a prospective study on septic shock that patients whose mottling score improved had a better prognosis that patients whose mottling score did not (14-day mortality 23% vs. 88%, *P* = 0.004) [5]. We measured mean skin perfusion on the area on which we have previously computed the mottling score (Figure 1). The area of interest, the distance between the camera and the skin, was standardized. Interestingly, we found a close relationship between changes of mean skin perfusion and changes of mottling score. In all the patients whose mottling score increased, the mean skin perfusion decreased, in patients whose mottling score decreased, the mean skin perfusion decreased, and finally in patients whose mottling score did not change, the mean perfusion also remained stable. It is the first direct evidence that mottling extension was related to skin perfusion and cutaneous microcirculation impairment.

**Figure 4:**
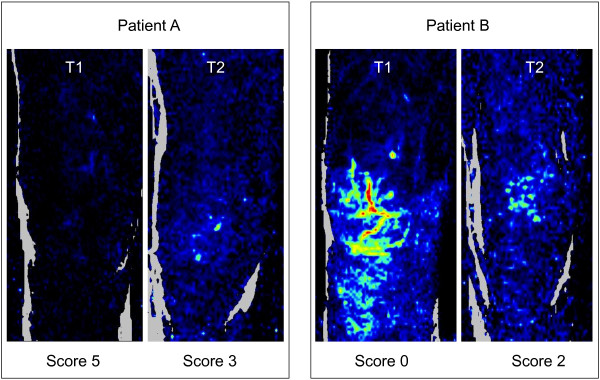
**Examples of laser Doppler images. A** mottling score decreased (improved), whereas in **B** mottling score increased (worsened).

During septic shock, microvascular dysfunction is heterogeneous, because it does not alter all of the organs or all of the territories of every organ [17,18]. Mottling preferentially develops around the knee like area of heterogeneous discoloration. Interestingly, laser Doppler imager confirmed heterogeneity of skin perfusion on the thigh (Figure 4). Furthermore, laser Doppler mapping showed that color-encoded signal was more important on the knee surface area compared with other leg surfaces (Figure 4), suggesting that vascular density or blood flow are more important in this area.

Our study has several limitations. It is a monocentric study and results need to be confirmed in a larger population. Nevertheless, although the size of this preliminary study was rather limited, it was sufficient to highlight significant results. Moreover, perfusion indices were calculated by the laser Doppler imager software and the physician who analyzed the data was blind to the mottling score and therefore was not influenced by the result of the clinical observation. Skin temperature was not measured in the study, and we cannot exclude variations of skin temperature between T1 and T2 that could affect microvascular skin blood flow. Finally, we explored global microvascular blood flow, but we did not analyze the precise mechanisms that lead to skin perfusion changes. Several intricate mechanisms, such as intravascular coagulation, increased leucocyte adhesion, or vascular tone modifications [18], potentially participated to the changes of microvascular perfusion.

## Conclusions

Using a laser Doppler imager, we have shown in patients admitted for septic shock that mottling score was related to skin perfusion. Moreover, we have shown that mottling score variations during resuscitation were related to skin perfusion changes providing additional evidence that mottling is the clinical expression of skin hypoperfusion.

## Competing interests

The authors had no competing of interest.

## Authors’ contribution

Conception and design: HAO, SB, BG, EM ; Analysis and interpretation: HAO, MA, AG, JLB, EM, BG ; Drafting the manuscript for important intellectual content: HAO, MA, AG, JLB, DM, NB, GO, EM, BG. All authors read and approved the final manuscript.
